# Enhancing the Nutritional Composition and Phenolic Compound Content of Sprouted Chickpeas Using Sucrose and Chitosan as Elicitors

**DOI:** 10.3390/molecules30081775

**Published:** 2025-04-15

**Authors:** Alejandra Linares-Castañeda, Luis Jorge Corzo-Ríos, Ana Elena Cedillo-Olivos, Xariss M. Sánchez-Chino, Rosalva Mora-Escobedo, Cristian Jiménez-Martínez

**Affiliations:** 1 Departamento de Ingeniería Bioquímica, Escuela Nacional de Ciencias Biológicas, Instituto Politécnico Nacional (IPN), Av. Wilfrido Massieu S/N, Unidad Profesional Adolfo López Mateos, Zacatenco, Alc. Gustavo A. Madero, C.P., Mexico City 07738, Mexico; 2 Unidad Profesional Interdisciplinaria de Biotecnología, Instituto Politécnico Nacional (IPN), Av. Acueducto S/N, Barrio La Laguna, Col. La Laguna Ticomán, Mexico City 07340, Mexico; 3 SECIHTI, Departamento de Salud, El Colegio de la Frontera Sur-Villahermosa, Carretera Federal Villahermosa-Reforma Km 15.5, Ra. Guineo Segunda Sección, C.P., Villahermosa 86280, Mexico

**Keywords:** chickpea, sprouting, elicitors, chitosan, sucrose

## Abstract

The use of elicitors during germination is a strategy to enhance the nutritional quality and biofunctional properties of various legumes, such as chickpeas, which are important sources of proteins and bioactive compounds. The objective of this study was to evaluate the effect of the use of chitosan (CH) and sucrose (SU) during sprouting on protein content, in vitro protein digestibility (IVPD), total phenolic content (TPC), and antioxidant activity (AOX). For this purpose, soaking time, elicitor concentration (CH or SU), and sprouting time were optimized to obtain maximum values for the response variables. The results showed that the optimal conditions for achieving increases in nutritional and biofunctional properties were 1 h of soaking, 0.35% *w*/*v*, and 5 days of sprouting for CH, and 2.55 h of soaking, 1% *w*/*v*, and 5 days of sprouting for SU. Under these conditions, protein content increased by 7–12%, IVPD by 78–86%, TPC by 379–327%, and AOX by 115% for CH and SU, respectively. Additionally, morphological changes were observed in the cellular structure of chickpea cotyledons, but no changes were detected in the crystalline structure of starch. These results contribute to the understanding of the effect of CH and SU in modifying the nutritional and biofunctional properties of chickpeas.

## 1. Introduction

Chickpea (*Cicer arietinum* L.) is the third-most cultivated legume in the world after soybean and bean. It belongs to the Leguminosae family. This legume contains significant amounts of protein (18–25%), carbohydrates (40–50%), lipids (4–6%), dietary fiber (25–27%), and minerals (3–4%) such as phosphorus, zinc, magnesium, calcium, and iron. It also provides essential vitamins, including riboflavin, niacin, thiamin, and folate [1,2,3,4,5]. Furthermore, it contains phenolic compounds such as catechin, chlorogenic acid, coumaric acid, ferulic acid, rutin, kaempferol 3-glucoside, cyanidin 3-glucoside, and quercetin; however, the content of phenolic compounds in chickpeas (1.44 mg GAE/g) is lower compared to in other legumes such as lentils (7.34 mg GAE/g) [6,7]. Additionally, chickpea contains bioactive compounds with antioxidant activity that may help prevent chronic diseases such as cardiovascular diseases, obesity, and cancer [8,9].

For consumption, legumes can be processed using various methods, including extractive, thermal, and biological treatments [10]. Germination is an effective biological method that enhances the nutritional value of seeds by improving protein digestibility and increasing bioactive compounds, such as polyphenols, which act as antioxidants in foods [11,12].

Germination is a complex process involving physiological and biochemical changes, including water absorption, changes in subcellular structure, root and sprout growth, the formation of the enzyme system, and the degradation of or changes in storage substances [12]. The germination conditions of legumes influence a series of morphological, physiological, and biochemical characteristics of plants and the content of phytochemicals, such as phenolic compounds. These compounds play an important role in seed dispersal and plant defense responses [13].

On the other hand, elicitors are substances that are applied in small quantities to activate transcriptional factors in seeds, regulating the expression of genes related to the production of secondary metabolites [14]. Elicitors promote the synthesis of antioxidant compounds due to the activation of the enzyme phenylalanine ammonia-lyase, which is involved in the biosynthesis of phenolic compounds [13].

Among the most used elicitors are molecules such as salicylic acid, chitosan, glutamic acid, hydrogen peroxide, and sucrose, along with treatments such as UV-B radiation, hydric stress, and ultrasound [15,16,17]. However, it is crucial to identify the type of elicitor, its concentration, and the duration of contact to ensure that it does not negatively impact seed germination.

This study evaluated the effect of elicitor concentration, specifically chitosan (CH) and sucrose (SU), and soaking and sprouting times on protein content, in vitro protein digestibility (IVPD), total phenolic content (TPC), and antioxidant activity (AOX) in chickpea. CH concentrations ranged from 0.1% to 0.5% (*w*/*v*), while SU concentrations ranged from 1% to 3% (*w*/*v*), soaking time ranged from 1 to 3 h, and sprouting time ranged from 1 to 5 days.

## 2. Results and Discussion

### 2.1. Protein Content

**Figure 1:**
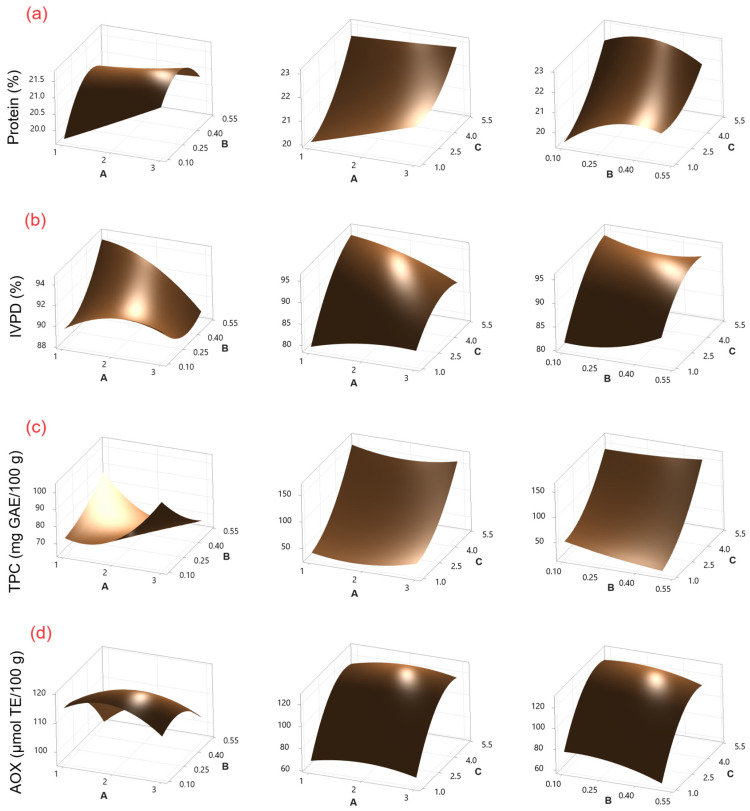
Surface response plots for (**a**) protein content, (**b**) IVPD, (**c**) TPC, and (**d**) AOX in chickpea sprouts treated with CH as an elicitor. Experimental factors: A = soaking time (h); B = CH concentration (% *w*/*v*); and C = sprouting time (days).

**Figure 2:**
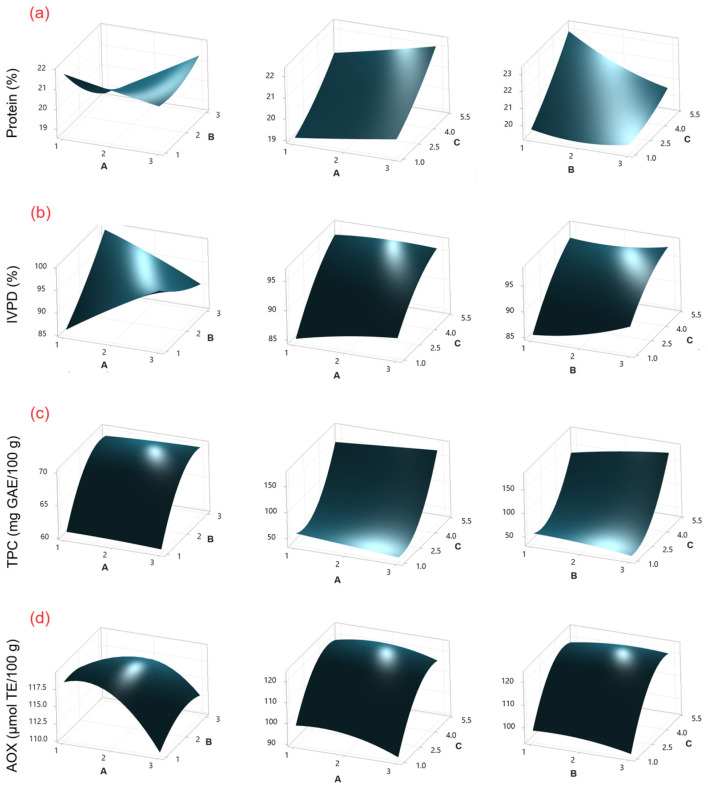
Surface response plots for (**a**) protein content, (**b**) IVPD, (**c**) TPC, and (**d**) AOX in chickpea sprouts treated with SU as an elicitor. Experimental factors: A = soaking time (h); B = SU concentration (% *w*/*v*); and C = sprouting time (days).

The protein content of chickpeas sprouted using CH as an elicitor ranged from 18.97 to 23.36 g/100 g (Table 1), while that of chickpeas treated with SU ranged from 18.08 to 23.52 g/100 g (Table 2). According to the ANOVA (Tables S1 and S5), sprouting time (C) was the factor that presented the most significant effect (*p* ≤ 0.05) on protein content for both elicitors. This effect can be observed in Pareto charts (Figures S1 and S2). On the other hand, the response surface plots (Figure 1a and Figure 2a) show that the protein content increases with increasing germination time. Similar protein content values have been reported in sprouted chickpeas without the use of elicitors (20.95–24.2 g/100 g) [2].

The application of CH and SU as elicitors to chickpeas has not been documented; however, Peñas et al. [18] reported that using chitosan as an elicitor during lentil sprouting had no significant effects compared to sprouting with water after eight days (~27 g/100 g). Similarly, it has been reported that protein content remained similar with and without the use of sucrose as an elicitor in sprouted buckwheat over ten days [19]. Based on these results and statistical analysis, it was observed that at the studied concentrations, CH and SU did not have a significant effect on protein content during seed sprouting. On the other hand, it has been reported that with an increase in sprouting time, protein content increased in chickpeas (~20–23 g/100 g), which may be attributed to protein synthesis from low-molecular-weight peptides [12,20,21].

### 2.2. In Vitro Protein Digestibility (IVPD)

The use of CH and SU as elicitors during germination has been studied in chickpeas, lentils, and mung beans [13,22,23]. However, changes in protein digestibility during the process were not investigated in the studies. In this study, IVPD values for CH ranged from 78.63% to 96.82% (Table 1), and those for SU ranged from 82.36% to 99.51% (Table 2). According to the ANOVA (Tables S2 and S6), for CH, the factors sprouting time (C), sprouting time–sprouting time (CC), and soaking time–sprouting time (AC) showed a significant effect (*p* ≤ 0.05), while for SU, the factors sprouting time (C) and soaking time–sucrose concentration (AB) were significant. This effect can be observed in Pareto charts (Figures S1 and S2). On the other hand, response surface plots (Figure 1b and Figure 2b) show that IVPD increases with increasing germination time. The results are similar to those reported by Khattak et al. [24] (~80% IVPD on day 5), and by Khalil et al. [21] (95% IVPD on day 4) for chickpea sprouting.

It has been reported that germination increases protein digestibility [12]. This may be related to protein hydrolysis during germination, which facilitates the release of amino acids from the protein structure, making them more digestible and absorbable in the body. Additionally, a reduction in antinutritional factors, such as protease inhibitors and phytic acid, has been observed, leading to high IVPD values, which are considered indicators of high-quality proteins [4,25,26].

### 2.3. Total Phenolic Content (TPC)

The TPC concentration for CH ranged from 39.18 to 175.10 mg GAE/100 g (Table 1), while for SU, it ranged from 45.45 to 180.69 mg GAE/100 g (Table 2). According to the ANOVA (Tables S3 and S7), for CH and SU, the factors of sprouting time (C) and the interaction of sprouting time–sprouting time (CC) showed a significant effect (*p* ≤ 0.05). Additionally, for SU, sucrose concentration–sprouting time (BC) was also significant. This effect can be observed in Pareto charts (Figures S1 and S2). On the other hand, TPC increases with increasing germination time as shown by the response surface plots (Figure 1c and Figure 2c). The resulting values are lower than those reported by Mesfin et al. [27] (272.5 mg GAE/100 g) after three days of chickpea sprouting without the use of elicitors.

Peñas et al. [18] reported that 50 ppm of CH significantly increased TPC in sprouted lentils over eight days (from ~3 to 3.9 mg GAE/g with CH). Similarly, Yu et al. [13] reported that SU concentrations of 2% and 3% increased TPC by up to 28% in sprouted mung beans. Additionally, it has been reported that 3% SU increased the total polyphenol content by 54% in buckwheat [28].

The increase in TPC may be attributed to the activation of endogenous hydrolases, such as phenylalanine ammonia-lyase, a key enzyme in phenol synthesis [29]. Additionally, during germination, the degradation of cell wall components occurs, releasing bound phenolic compounds [30].

With respect to elicitors, CH and SU can influence the germination of legumes by modulating key metabolic pathways, including the phenylpropanoid pathway and the pentose phosphate pathway. The phenylpropanoid pathway is essential for the production of secondary metabolites such as phenolic compounds, which play an important role in the stress response, and in the activation of plant defense mechanisms during germination [14,18]. The elicitors, used alone or in combination, improve germination efficiency and stress resilience in legumes. Further transcriptomic and metabolomic studies could precisely map these interactions.

### 2.4. Antioxidant Activity (AOX)

AOX values for CH ranged from 64.92 to 120.65 μmol TE/100 g, while for SU, they ranged from 91.08 to 120.1365 μmol TE/100 g. According to the ANOVA (Tables S4 and S8), for CH, the factors sprouting time (C), sprouting time–sprouting time (CC), and chitosan concentration (B) were significant (*p* ≤ 0.05), while for SU, the factors sprouting time (C), sprouting time–sprouting time (CC), soaking time (A), and soaking time–soaking time (AA) were significant. This effect can be observed in Pareto charts (Figures S1 and S2). On the other hand, the response surface plots (Figure 1d and Figure 2d) show that AOX increases with increasing germination time. The results are similar to those reported by Linares-Castañeda et al. [1] (121.18 μmol TE/100 g) in sprouted chickpeas treated with hydrogen peroxide as an elicitor after five days of sprouting.

Additionally, Mendoza-Sánchez et al. [31] reported that 7 µM CH increased antioxidant capacity in germinated beans by up to 30%. Likewise, Yu et al. [13] demonstrated that SU (1%, 2%, and 3%) increased antioxidant properties by 38% in sprouted mung beans.

Antioxidant activity can be attributed to the increase in phenolic compounds during sprouting and elicitation because elicitors stimulate the pentose phosphate and phenylpropanoid pathways. These compounds can neutralize excess free radicals and maintain intracellular balance in the body, thereby exerting protective effects against oxidative stress [28,32].

### 2.5. Optimization and Validation of the Sprouting and Elicitation Process

The four response variables were maximized for each elicitor (Figures S3 and S4), obtaining the optimal conditions for sprouting and elicitation (Table 3). The optimal sprouting conditions using CH as an elicitor were 1 h of soaking in 0.35% *w*/*v* and 5 days of sprouting (D = 0.92). Meanwhile, for SU as an elicitor, the optimal conditions were 2.55 h of soaking in 1% *w*/*v* and 5 days of sprouting (D = 0.89). Subsequently, chickpeas were sprouted under these optimized conditions, and the corresponding flours were obtained to validate the predicted values. The results showed that the experimentally obtained values were similar to those predicted by the model (Table 4). The use of CH increased IVPD by 78%, TPC by 379%, and AOX by 115% compared to raw seeds. Additionally, it increased IVPD by 14%, TPC by 39%, and AOX by 1% compared to CCH. On the other hand, the use of SU increased protein content by 12%, IVPD by 86%, TPC by 327%, and AOX by 115% compared to raw seeds. Additionally, it increased protein content by 9%, IVPD by 8%, TPC by 9%, and AOX by 1% compared to CSU.

#### 2.5.1. Proximate Chemical Analysis

During germination and elicitation, macronutrients such as proteins, lipids, and carbohydrates serve as essential energy sources for seed growth, resulting in changes in chemical composition [33,34]. According to the results in Table 5, lipid content ranged from 6.69 to 8.27 g/100 g. Similar values (6–8 g/100 g) have been reported by Khalil et al. [21] in sprouted chickpeas. Additionally, lipid content significantly increased (*p* ≤ 0.05) by 24% with CH and 15% with SU compared to raw seeds. With the addition of the elicitor, an increase of 12% with CH and 2% with SU was observed compared to the control treatments (CCH and CSU), respectively. In seeds, storage lipids are metabolized to provide the necessary energy for germination, which can lead to a decrease in lipid content. However, an increase can be attributed to the synthesis of structural lipids (such as phospholipids) for the formation of new membranes [11,35].

For crude fiber (Table 5), values ranged from 1.16 to 2.11 g/100 g. Khalil et al. [21] reported higher crude fiber values (4–6 g/100 g) in sprouted chickpeas. As observed, fiber content decreased by 40% when CH was used as an elicitor and by 6% with SU compared to raw seeds. Meanwhile, in the control treatments, fiber content increased by 45% with CH and 3% with SU. A 55% reduction in fiber content has been reported during sprouting in pigeon pea, indicating that the effects of sprouting and elicitor use depend on the type of legume studied [36,37]. Additionally, the reduction in crude fiber may be attributed to enzyme activity (such as cellulases, hemicellulases, and pectinases), which degrades the cell wall [38,39].

Ash content ranged from 2.74 to 3.16 g/100 g. Similar ash values (2–3 g/100 g) have been reported in sprouted chickpeas [21]. In this study, an increase of up to 10% in ash content was observed when SU and CSU were used, compared to raw seeds and the other two treatments. SU and CSU involved a longer soaking time (2.55 h) compared to CH and CCH (1 h). This increase may be attributed to increased phytase activity, releasing minerals bound to protein-based compounds [11].

NFE content ranged from 64.32 to 67.93 g/100 g. Khalil et al. [21] reported similar values (~60 g/100 g) in sprouted chickpeas. As observed in Table 5, the NFE content decreased by 3% in the presence of CH and by 10% when SU was used compared to raw seeds. Additionally, NFE content decreased by 3% with SU compared to CSU. These changes may be due to carbohydrate hydrolysis, as chickpeas are primarily composed of starch, which is hydrolyzed by enzymes (α-amylase, β-amylase, and maltase) into oligosaccharides, disaccharides, and monosaccharides for various biochemical activities during germination [28,37].

#### 2.5.2. Confocal Laser Scanning Microscopy (CLSM)

**Figure 3:**
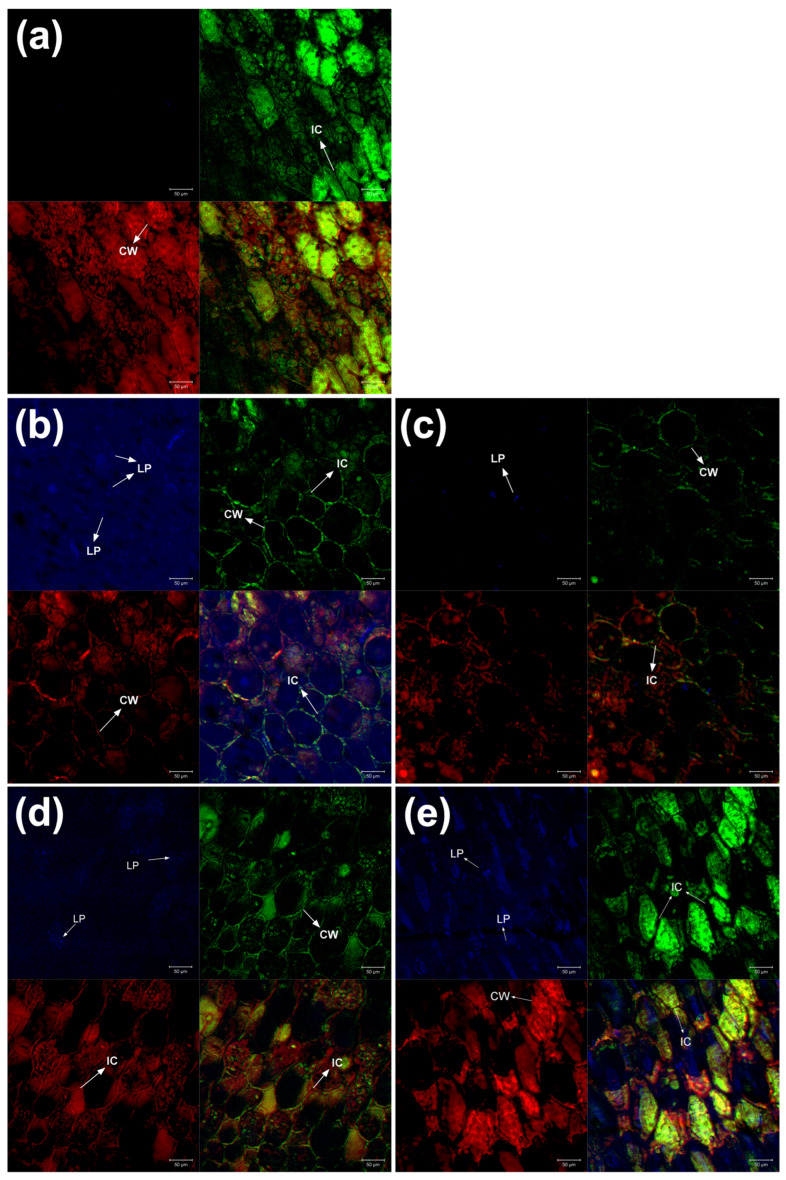
CLSM images (20X) of chickpea cotyledons. Fluorescent markers: Rhodamine (red), FITC (green), and Auramine O (blue). (**a**) Raw, (**b**) CH, (**c**) CCH, (**d**) SU, and (**e**) CSU. Abbreviations: CW: cell wall; IC: intercellular space; LP: lipid body.

In addition to the nutritional and bioactive compound changes occurring during seed germination, structural changes also occur in cotyledon cells [11]. Through CLSM, cell walls, intercellular spaces, lipid bodies, carbohydrates, and structural proteins were identified in the analyzed samples. Generally, differences were observed in cotyledon cells between raw samples and samples treated with CH, CCH, SU, and CSU (Figure 3). It has been reported that germination induces changes in cell wall structure and the cytoplasmic matrix [40].

The plant cell wall consists of several polysaccharides (cellulose, hemicelluloses, and pectins), as well as structural proteins and phenolic compounds [41]. The cotyledon cell sizes in the samples were approximately 50 μm, and similar cell sizes have been reported in bean cotyledons [42].

In the raw sample (Figure 3a), slightly compact and ellipsoid-shaped cell structures were observed. Chickpeas have been reported to exhibit this type of ellipsoidal cell [43]. In contrast, in the CH sample (Figure 3b), irregularly shaped lipid bodies were observed, as well as semi-spherical cells and an increase in intercellular spaces. In the CCH sample (Figure 3c), very few lipid bodies and semi-spherical cells were identified. In the SU sample (Figure 3d), hexagon-shaped cell structures were observed, and no lipid bodies were detected in the analyzed region. Regarding CSU (Figure 3e), the cells showed an ellipsoidal shape with an increase in intercellular spaces.

Structural changes in cotyledon cells may be due to the enzymatic degradation of the cell wall induced by germination [44]. It has been reported that germination increases intercellular spaces [11] and results in loosely packed cells [45]. Additionally, the low presence of lipids may be due to the naturally low lipid content in legume cotyledons [46].

Changes in cellular structures can impact the bioavailability of certain nutrients. An increase in intercellular spaces and alterations to the cell wall can enhance the release and absorption of these nutrients. Additionally, the concentration of some bioactive compounds, like phenolic compounds, may increase as they are released from the matrices in which they are typically found [47].

#### 2.5.3. X-Ray Diffraction (XRD)

**Figure 4:**
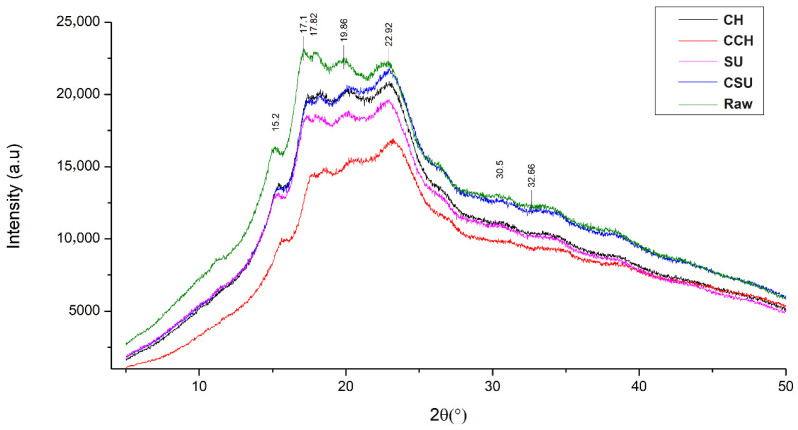
XRD of raw and sprouted and elicited samples. Abbreviations: CH = chitosan; CCH = control chitosan; SU = sucrose; CSU = control sucrose.

Figure 4 shows the XRD pattern of the raw sample and the sprouted and elicited samples. The intensity of sample peaks decreased after germination, which has also been reported by Kaur and Prasad [48] in sprouted chickpeas. The analysis showed a type C pattern (a combination of types A and B), which is characteristic of legume starches [48]. Additionally, no changes were observed in peak positions, which were around 15°, 17°, 18°, 20°, 23°, 31°, and 33°. Similar patterns have been reported in chickpeas [49] and sprouted peas [50].

XRD patterns serve as an analytical technique to evaluate the crystalline and amorphous nature of compounds present in flour samples [50]. Sharp peaks represent crystalline regions, while diffuse peaks represent the amorphous region [4].

Furthermore, relative crystallinity in the raw sample was 64.78% and decreased to 50.48% in CH. However, in CCH, SU, and CSU, relative crystallinity increased to 65.56%, 68.23%, and 66.24%, respectively. These different degrees of crystallinity indicate differences in the chemical structure and composition of starches. During germination, starch granule hydrolysis occurs in a non-uniform manner, with amorphous regions being hydrolyzed first, followed by crystalline regions. As a result, crystallinity index values depend on the specific region being hydrolyzed, which may lead to either an increase or decrease in starch granule crystallinity [51].

For example, a decrease in the crystallinity index has been reported in sprouted pea [50] and chickpea flours, attributed to the reduction in crystalline regions due to starch granule hydrolysis by amylases produced during germination [4]. The breakdown of protein structures also reduces the α-helix structure, leading to an increase in a disordered structure [45]. However, Kaur and Prasad [48] reported an increase in crystallinity in mung beans during 12 h of sprouting.

## 3. Materials and Methods

### 3.1. Biological Material and Reagents

Chickpea seeds were purchased from the local market, originating from the state of Sinaloa, Mexico. The seeds were selected by removing foreign materials and damaged seeds. Likewise, 100 g of chickpea was used for each experimental treatment.

Chitosan with a medium molecular weight (190,000–310,000 Da) and a degree of deacetylation between 75 and 85%, sucrose, Folin–Ciocalteu reagent, 2,2-diphenyl-1-picrylhydrazyl (DPPH), 6-hydroxy-2,3,7,8-tetramethylchroman-2-carboxylic acid (TROLOX), gallic acid, Bradford reagent, pepsin (E.C. 3.4.23.1, PP-77163, 800–2500 units/mg protein, from pig stomach), and pancreatin (P-1750, 4X USP, from pig pancreas) were purchased from Sigma-Aldrich (St. Louis, MO, USA). Methanol was obtained from Reasol (Mexico City, Mexico). All other chemicals were of analytical grade and sourced from JT Baker (Phillipsburg, NJ, USA).

### 3.2. Experimental Design

To optimize the sprouting and elicitation process, Box–Behnken designs with four central points were used. The evaluated experimental factors were soaking time (A), elicitor concentration (B), and sprouting time (C), as described in Table 1 and Table 2. The response variables were protein content, IVPD, TPC, and AOX.

#### Sprouting and Elicitation of Chickpea

The selected seeds were disinfected with 0.5% *v*/*v* sodium hypochlorite (NaClO), and then rinsed with purified water to remove residual NaClO. The elicitation treatment was applied to the seeds during imbibition, following the specified soaking time (1–3 h) and concentration of each elicitor (0.1–0.5% *w*/*v* for CH and 1–3% *w*/*v* for SU), with agitation every 15 min. Germination was carried out at 28 °C and 70% relative humidity until the sprouting time (1–5 days) was completed for each treatment. Finally, the samples were dried at 50 °C for 24 h and then milled and sieved through a 60-mesh sieve (250 μm) to obtain the flour.

##### In Vitro Protein Digestibility (IVPD)

The methodology established by Linares-Castañeda et al. [1] was used. The IVPD on a dry matter basis was estimated as follows:

(1) $$IVPD\%=\frac{initial\;protein-final\;non\;digested\;protein}{initial\;protein}\times 100$$

##### Extraction and Determination of Total Phenolic Content (TPC)

Extracts were prepared in aqueous methanol (80% *v*/*v*) containing HCl (pH 2.8). The extraction of total phenolics was carried out for 16 h in darkness (4 °C), followed by centrifugation at 4000 g for 20 min, and the supernatant was collected. Total phenolic content was estimated using the Folin–Ciocalteau method, using gallic acid as the standard, and absorbance was measured at 765 nm [52]. Results are expressed as mg gallic acid equivalent/100 g dry matter (mg GAE/100 g DM).

##### Antioxidant Activity (AOX)

The analysis was performed according to the method described by Brand-Williams et al. [53] adapted to a multiwell plate. The AOX of the extract was evaluated using the stable radical DPPH in methanolic solution and Trolox for the standard curve. After 30 min of incubation in the dark, absorbance was measured at 517 nm. Results are expressed in μmol Trolox equivalents per 100 g of dry matter (μmol TE/100 g DM).

### 3.3. Optimization and Validation

Based on the results from the Box–Behnken designs, the sprouting process was optimized for elicitation with chitosan and sucrose; all four study variables were maximized. The predicted optimal conditions were experimentally validated for CH and SU. Additionally, a germination control was established for each elicitor using purified water under the same soaking and germination conditions, that is, 1 h of soaking in purified water and 5 days of germination for the chitosan control (CCH) and 2.55 h of soaking in purified water and 5 days of germination for the sucrose control (CSU), while the raw seed was also analyzed (Table 3).

#### 3.3.1. Proximate Composition

The moisture, crude protein, lipids, crude fiber, and ash content of chickpea flour samples were determined using the standard Association of Official Analytical Chemists (AOAC) method [54]. The nitrogen-free extract (NFE) was measured using the difference method. All the results are reported as g/100 g of dry matter (g/100 g DM).

#### 3.3.2. Confocal Laser Scanning Microscopy (CLSM)

The microstructure of chickpea cotyledons was investigated using CLSM according to the methodology described by Linares-Castañeda et al. [1].

#### 3.3.3. X-Ray Diffraction (XRD)

The samples were analyzed using a Rigaku MiniFlex diffractometer (Rigaku Holdings Corporation, Tokyo, Japan) to obtain X-ray diffraction patterns with Cu Kα radiation (λ = 1.54056 Å). The results of the XRD analysis were the 2θ diffraction angles, intensity, and d-spacing. The relative percentage of crystallinity of the samples was determined from the area between the crystalline and amorphous regions in the XRD patterns using OriginPro 8.1 software (OriginLab, Northampton, MA, USA).

### 3.4. Statistical Analysis

Analysis of variance (ANOVA) was conducted (*p* ≤ 0.05), and Tukey’s test was used to determine significant differences at *p* ≤ 0.05. Minitab software version 21.2 (Minitab Inc., State College, PA, USA) was used.

## 4. Conclusions

The findings of this study demonstrate that CH and SU are effective elicitors in enhancing the nutritional and functional properties of chickpea sprouts.

The results demonstrated that both elicitors, under optimal conditions, significantly increased IVPD, TPC, and AOX compared to raw seeds. The protein content was found to be significantly increased only when SU was used as an elicitor. The optimal conditions for CH were 1 h of soaking, a concentration of 0.35% *w*/*v*, and 5 days of sprouting, while for SU, the optimal conditions were 2.55 h of soaking, a concentration of 1% *w*/*v*, and 5 days of sprouting. These conditions allowed for the maximum values of protein (22.10–23.00%), IVPD (92.30–96.58%), TPC (163.15–145.44 mg GAE/100 g), and AOX (120.32–120.62 μmol TE/100 g). Additionally, the use of elicitors can modify the cellular morphology of chickpea cotyledons and the relative crystallinity of the starch.

CH and SU treatments provide a cost-effective and scalable method for producing nutrient-rich chickpea sprouts for functional foods, dietary supplements, or plant protein ingredients. The increase in phenolic compounds and improved protein digestibility may help manage chronic diseases by enhancing antioxidant activity and metabolic health.

This work bridges agricultural innovation and nutritional science, providing actionable strategies to improve legume-based foods in the face of global health challenges. Using elicitors like CH and SU aligns with sustainable food production goals, leveraging natural compounds to maximize crop value without genetic modification.

This study advances the understanding of legume biofortification by optimizing germination conditions with chitosan and sucrose. It delivers a practical, eco-friendly solution to enhance the dietary quality of chickpeas—a crop critical for food security and nutrition.

## Figures and Tables

**Table 1:** Box–Behnken matrix or analysis of parameters: protein content, in vitro protein digestibility (IVPD), total phenolic content (TPC), and antioxidant activity (AOX) of chitosan-elicited chickpeas.

ST–CC–SD	Protein ^1^	IVPD ^2^	TPC ^3^	AOX ^4^
3–0.3–5	23.36 ^a^	89.32 ^e^	160.58 ^b^	119.10 ^a^
1–0.3–1	19.73 ^g^	78.63 ^i^	44.33 ^g^	69.30 ^f^
2–0.1–1	18.97 ^h^	83.19 ^g^	39.55 ^g^	71.62 ^f^
3–0.1–3	21.01 ^d^	88.58 ^f^	123.87 ^d^	116.19 ^c^
2–0.5–1	20.87 ^e^	84.24 ^g^	39.18 ^g^	64.92 ^h^
1–0.3–5	22.15 ^b^	95.19 ^b^	175.10 ^a^	119.07 ^a^
3–0.3–1	22.07 ^c^	82.89 ^h^	40.57 ^g^	66.55 ^g^
2–0.1–5	22.24 ^b^	96.82 ^a^	134.06 ^c^	120.65 ^a^
3–0.5–3	20.06 ^g^	90.04 ^d^	63.20 ^f^	100.42 ^d^
2–0.5–5	22.53 ^b^	92.39 ^c^	170.62 ^a^	119.98 ^a^
1–0.1–3	20.56 ^f^	88.66 ^f^	78.72 ^e^	118.00 ^a^
1–0.5–3	20.89 ^e^	96.80 ^a^	67.55 ^f^	90.94 ^e^
2–0.3–3 *	21.28 ^d^	90.69 ^d^	68.32 ^f^	117.15 ^b^

* Mean values of four central points. Units: 1 = g/100 g; 2 = %; 3 = mg GAE/100 g; 4 = μmol TE/100 g. All determinations were made on a dry matter (DM) basis. Abbreviations: ST = soaking time (h); CC = chitosan concentration (% *w*/*v*); SD = sprouting time (days). Different superscript letters (a–i) within the same column indicate a significant difference (Tukey’s test, *p* ≤ 0.05).

**Table 2:** Box–Behnken matrix or analysis of parameters: protein content, in vitro protein digestibility (IVPD), total phenolic content (TPC), and antioxidant activity (AOX) of sucrose-elicited chickpeas.

ST–SC–SD	Protein ^1^	IVPD ^2^	TPC ^3^	AOX ^4^
2–3–5	22.03 ^b^	97.29 ^b^	180.69 ^a^	120.05 ^a^
1–1–3	22.01 ^b^	82.36 ^f^	69.25 ^e^	118.41 ^b^
3–1–3	21.48 ^b^	99.51 ^a^	61.20 ^f^	108.31 ^e^
3–2–5	21.28 ^c^	93.22 ^c^	176.30 ^b^	119.92 ^a^
3–3–3	21.06 ^c^	94.15 ^c^	61.42 ^f^	112.77 ^d^
1–3–3	18.08 ^g^	97.76 ^b^	68.53 ^e^	116.41 ^c^
1–2–1	20.02 ^e^	88.06 ^d^	52.78 ^g^	96.50 ^g^
2–1–1	18.44 ^g^	85.87 ^e^	51.70 ^g^	100.21 ^f^
2–3–1	19.39 ^f^	88.36 ^d^	45.99 ^h^	94.73 ^h^
1–2–5	20.55 ^d^	97.94 ^a^	153.51 ^c^	120.05 ^a^
2–1–5	23.52 ^a^	98.16 ^a^	138.41 ^d^	120.13 ^a^
3–2–1	20.38 ^e^	85.79 ^e^	45.45 ^h^	91.08 ^i^
2–2–3 *	20.33 ^e^	93.04 ^c^	68.10 ^e^	118.82 ^b^

* Mean values of four central points. Units: 1 = g/100 g; 2 = %; 3 = mg GAE/100 g; 4 = μmol TE/100 g. All determinations were made on a dry matter (DM) basis. Abbreviations: ST = soaking time (h); SC = sucrose concentration (% *w*/*v*); SD = sprouting time (days). Different superscript letters (a–i) within the same column indicate a significant difference (Tukey’s test, *p* ≤ 0.05).

**Table 3:** Optimization of experimental designs.

Optimal Conditions				
Elicitor	CH	CCH	SU	CSU
Soaking time (h)	1	1	2.55	2.55
Elicitor concentration (% *w*/*v*)	0.35	-	1	-
Sprouting time (days)	5	5	5	5
Protein adjustment (%)	22.78		23.03	
IVPD adjustment (%)	96.02		99.50	
TPC adjustment (mg GAE/100 g sample)	168.76		148.36	
AOX adjustment (μmol TE/100 g sample)	115.96		117.48	
Desirability value (D)	0.92		0.89	

Abbreviations: IVPD = in vitro protein digestibility; TPC = total phenolic content; AOX = antioxidant activity; CH = chitosan; CCH = control chitosan; SU = sucrose; CSU = control sucrose.

**Table 4:** Experimental validation of sprouted and elicited samples with chitosan and sucrose.

Response Variable	Raw	CH	CCH	SU	CSU
Protein ^1^	20.59 ± 0.86 ^c^	22.10 ± 0.52 ^b^	21.92 ± 0.24 ^b^	23.00 ± 0.27 ^a^	21.14 ± 0.24 ^b^
IVPD ^2^	51.81 ± 0.99 ^e^	92.30 ± 1.57 ^b^	81.07 ± 2.78 ^d^	96.58 ± 2.54 ^a^	89.40 ± 1.95 ^c^
TPC ^3^	34.06 ± 1.03 ^e^	163.15 ± 3.66 ^a^	117.77 ± 4.24 ^d^	145.44 ± 2.80 ^b^	133.01 ± 2.20 ^c^
AOX ^4^	56.03 ± 0.16 ^c^	120.32 ± 0.09 ^a^	119.15 ± 0.58 ^b^	120.63 ± 0.32 ^a^	119.44 ± 0.41 ^b^

Data are presented as mean ± standard deviation (*n* = 3). Different superscript letters indicate significant differences (Tukey’s test, *p* ≤ 0.05). Abbreviations: IVPD = in vitro protein digestibility; TPC = total phenolic content; AOX = antioxidant activity; CH = chitosan; CCH = control chitosan; SU = sucrose; CSU = control sucrose. Units: 1 = g/100 g; 2 = %; 3 = mg GAE/100 g; 4 = μmol TE/100 g. All determinations were made on a dry matter (DM) basis.

**Table 5:** Proximate chemical analysis of raw, sprouted and elicited samples with CH and SU (g/100 g DM).

Chemical Composition	Raw	CH	CCH	SU	CSU
Protein	20.59 ± 0.86 ^c^	22.10 ± 0.52 ^b^	21.92 ± 0.24 ^b^	23.00 ± 0.27 ^a^	21.14 ± 1.76 ^b^
Lipids	6.69 ± 0.38 ^c^	8.27 ± 0.45 ^a^	7.37 ± 0.23 ^b^	7.70 ± 0.55 ^a^	7.54 ± 0.58 ^b^
Crude fiber	1.93 ± 0.25 ^a^	1.16 ± 0.13 ^c^	2.11 ± 0.20 ^a^	1.82 ± 0.42 ^b^	1.87 ± 0.12 ^a^
Ashes	2.86 ± 0.09 ^b^	2.80 ± 0.07 ^b^	2.74 ± 0.16 ^b^	3.16 ± 0.07 ^a^	3.13 ± 0.03 ^a^
NFE	67.93 ± 0.89 ^a^	65.67 ± 1.16 ^b^	65.86 ± 0.18 ^b^	64.32 ± 0.48 ^c^	66.32 ± 2.14 ^a^

Values represent mean ± standard deviation (*n* = 3). Different superscript letters indicate significant differences (Tukey’s test, *p* ≤ 0.05). Abbreviations: NFE = nitrogen-free extract; CH = chitosan; CCH = control chitosan; SU = sucrose; CSU = control sucrose.

## Data Availability

The data are available in the Supplementary Materials.

## Supplementary Materials

The following supporting information can be downloaded at https://www.mdpi.com/article/10.3390/molecules30081775/s1, Figures S1 and S2: Pareto charts for chitosan and sucrose as elicitors during chickpea sprouting. (a): Protein content; (b): in vitro protein digestibility (IVPD); (c): total phenolic content (TPC); and (d): antioxidant activity (AOX). A: Soaking time; B: chitosan concentration; and C: sprouting time. Figures S3 and S4: Optimal conditions for chitosan and sucrose as elicitors in chickpea germination. Tables S1–S8: Analysis of variance (ANOVA) for chitosan and sucrose across all studied variables. Tables S9 and S10: Statistical parameters used for the analysis of means by the Tukey test.
