# Supplementary material for: Enhancing the Nutritional Composition and Phenolic Compound Content of Sprouted Chickpeas Using Sucrose and Chitosan as Elicitors
Source: Molecules. 2025 Apr 15;30(8):1775. doi: 10.3390/molecules30081775 (PMC12029772; doi:10.3390/molecules30081775)
Supplement: Supplementary file 1 [file molecules-30-01775-s001.zip › molecules-3536769-supplementary.pdf]

Table S1. Analysis of Variance of Protein content for chitosan as elicitor.

Source	DF	Adj. SS	Adj. MS	F-value	P-value
Model	9	15.1898	1.68775	3.22	0.084
Linear	3	10.9064	3.63548	6.94	0.022
Soaking time (h)	1	1.2569	1.25695	2.40	0.172
Chitosan concentration (% w/v)	1	0.3067	0.30667	0.59	0.473
Sprouting time (days)	1	9.3428	9.34283	17.84	0.006
Square	3	2.9106	0.97021	1.85	0.238
Soaking time (h)* Soaking time (h)	1	0.0006	0.00064	0.00	0.973
Chitosan concentration (% w/v)*Chitosan concentration (% w/v)	1	1.7473	1.74729	3.34	0.118
Sprouting time (days)*Sprouting time (days)	1	1.1627	1.16271	2.22	0.187
2-Way interaction	3	1.3727	0.45756	0.87	0.505
Soaking time (h)*Chitosan concentration (% w/v)	1	0.4115	0.41155	0.79	0.409
Soaking time (h)*Sprouting time (days)	1	0.3185	0.31854	0.61	0.465
Chitosan concentration (% w/v)*Sprouting time (days)	1	0.6426	0.64260	1.23	0.310
Error	6	3.1413	0.52356		
Lack-of-fit	3	3.1067	1.03557	89.72	0.002
Pure error	3	0.0346	0.01154		
Total	15	18.3311			

Table S2. Analysis of Variance of *In vitro* protein digestibility (IVPD) for chitosan as elicitor.

Source	DF	Adj. SS	Adj. MS	F-value	P-value
Model	9	359.204	39.912	9.67	0.006
Linear	3	264.135	88.045	21.33	0.001
Soaking time (h)	1	8.925	8.925	2.16	0.192
Chitosan concentration (% w/v)	1	4.818	4.818	1.17	0.321
Sprouting time (days)	1	250.392	250.392	60.67	0.000
Square	3	50.765	16.922	4.10	0.067
Soaking time (h)* Soaking time (h)	1	5.375	5.375	1.30	0.297
Chitosan concentration (% w/v)*Chitosan concentration (% w/v)	1	8.899	8.899	2.16	0.192
Sprouting time (days)*Sprouting time (days)	1	36.491	36.491	8.84	0.025
2-Way interaction	3	44.303	14.768	3.58	0.086
Soaking time (h)*Chitosan concentration (% w/v)	1	11.110	11.110	2.69	0.152
Soaking time (h)*Sprouting time (days)	1	25.687	25.687	6.22	0.047
Chitosan concentration (% w/v)*Sprouting time (days)	1	7.505	7.505	1.82	0.226
Error	6	24.764	4.127		
Lack-of-fit	3	24.708	8.236	441.51	0.000
Pure error	3	0.056	0.019		
Total	15	383.967			

Table S3. Analysis of Variance of Total phenolic compounds for chitosan as elicitor.

Source	DF	Adj. SS	Adj. MS	F-value	P-value
Model	9	32671.9	3630.2	11.01	0.004
Linear	3	28631.3	9543.8	28.95	0.001
Soaking time (h)	1	63.3	63.3	0.19	0.677
Chitosan concentration (% w/v)	1	159.0	159.0	0.48	0.513
Sprouting time (days)	1	28409.0	28409.0	86.18	0.000
Square	3	3058.1	1019.4	3.09	0.111
Soaking time (h)* Soaking time (h)	1	590.9	590.9	1.79	0.229
Chitosan concentration (% w/v)*Chitosan concentration (% w/v)	1	32.7	32.7	0.10	0.764
Sprouting time (days)*Sprouting time (days)	1	2434.6	2434.6	7.39	0.035
2-Way interaction	3	982.5	327.5	0.99	0.457
Soaking time (h)*Chitosan concentration (% w/v)	1	612.6	612.6	1.86	0.222
Soaking time (h)*Sprouting time (days)	1	29.0	29.0	0.09	0.777
Chitosan concentration (% w/v)*Sprouting time (days)	1	340.9	340.9	1.03	0.348
Error	6	1977.9	329.6		
Lack-of-fit	3	1972.2	657.4	344.18	0.000
Pure error	3	5.7	1.9		
Total	15	34649.8			

Table S4. Analysis of Variance of Antioxidant activity for chitosan as elicitor.

Source	DF	Adj. SS	Adj. MS	F-value	P-value
Model	9	7196.21	799.58	28.02	0.000
Linear	3	5643.24	1881.08	65.93	0.000
Soaking time (h)	1	3.07	3.07	0.11	0.754
Chitosan concentration (% w/v)	1	315.05	315.05	11.04	0.016
Sprouting time (days)	1	5325.12	5325.12	186.63	0.000
Square	3	1510.07	503.36	17.64	0.002
Soaking time (h)* Soaking time (h)	1	133.38	133.38	4.67	0.074
Chitosan concentration (% w/v)*Chitosan concentration (% w/v)	1	99.46	99.46	3.49	0.111
Sprouting time (days)*Sprouting time (days)	1	1277.24	1277.24	44.76	0.001
2-Way interaction	3	42.89	14.30	0.50	0.695
Soaking time (h)*Chitosan concentration (% w/v)	1	31.89	31.89	1.12	0.331
Soaking time (h)*Sprouting time (days)	1	1.92	1.92	0.07	0.804
Chitosan concentration (% w/v)*Sprouting time (days)	1	9.08	9.08	0.32	0.593
Error	6	171.19	28.53		
Lack-of-fit	3	171.09	57.03	1654.54	0.000
Pure error	3	0.10	0.03		
Total	15	7367.40			

Table S5. Analysis of Variance of Protein content for sucrose as elicitor.

Source	DF	Adj. SS	Adj. MS	F-value	P-value
Model	9	20.1998	2.2444	1.83	0.238
Linear	3	15.0438	5.0146	4.08	0.067
Soaking time (h)	1	1.5632	1.5632	1.27	0.302
Sucrose concentration (% w/v)	1	2.9897	2.9897	2.43	0.170
Sprouting time (days)	1	10.4910	10.4910	8.54	0.027
Square	3	0.5536	0.1845	0.15	0.926
Soaking time (h)*Soaking time (h)	1	0.0014	0.0014	0.00	0.974
Sucrose concentration (% w/v)*Sucrose concentration (% w/v)	1	0.3801	0.3801	0.31	0.598
Sprouting time (days)*Sprouting time (days)	1	0.1720	0.1720	0.14	0.721
2-Way interaction	3	4.6024	1.5341	1.25	0.372
Soaking time (h)*Sucrose concentration (% w/v)	1	3.0780	3.0780	2.51	0.164
Soaking time (h)*Sprouting time (days)	1	0.0356	0.0356	0.03	0.870
Sucrose concentration (% w/v)*Sprouting time (days)	1	1.4889	1.4889	1.21	0.313
Error	6	7.3687	1.2281		
Lack-of-fit	3	7.0001	2.3334	18.99	0.019
Pure error	3	0.3686	0.1229		
Total	15	27.5685			

Table S6. Analysis of Variance of *In vitro* protein digestibility (IVPD) for sucrose as elicitor.

Source	DF	Adj. SS	Adj. MS	F-value	P-value
Model	9	330.956	36.773	3.45	0.073
Linear	3	207.840	69.280	6.51	0.026
Soaking time (h)	1	5.359	5.359	0.50	0.505
Sucrose concentration (% w/v)	1	17.033	17.033	1.60	0.253
Sprouting time (days)	1	185.447	185.447	17.41	0.006
Square	3	10.987	3.662	0.34	0.795
Soaking time (h)*Soaking time (h)	1	0.578	0.578	0.05	0.823
Sucrose concentration (% w/v)*Sucrose concentration (% w/v)	1	2.466	2.466	0.23	0.647
Sprouting time (days)*Sprouting time (days)	1	7.942	7.942	0.75	0.421
2-Way interaction	3	112.129	37.376	3.51	0.089
Soaking time (h)*Sucrose concentration (% w/v)	1	107.790	107.790	10.12	0.019
Soaking time (h)*Sprouting time (days)	1	1.500	1.500	0.14	0.720
Sucrose concentration (% w/v)*Sprouting time (days)	1	2.839	2.839	0.27	0.624
Error	6	63.893	10.649		
Lack-of-fit	3	63.478	21.159	153.12	0.001
Pure error	3	0.415	0.138		
Total	15	394.849			

Table S7. Analysis of Variance of Total phenolic compounds for sucrose as elicitor.

Source	DF	Adj. SS	Adj. MS	F-value	P-value
Model	9	32733.8	3637.1	72.18	0.000
Linear	3	25811.2	8603.7	170.76	0.000
Soaking time (h)	1	0.0	0.0	0.00	0.989
Sucrose concentration (% w/v)	1	162.6	162.6	3.23	0.123
Sprouting time (days)	1	25648.6	25648.6	509.04	0.000
Square	3	6119.5	2039.8	40.48	0.000
Soaking time (h)*Soaking time (h)	1	0.0	0.0	0.00	0.980
Sucrose concentration (% w/v)*Sucrose concentration (% w/v)	1	33.8	33.8	0.67	0.444
Sprouting time (days)*Sprouting time (days)	1	6085.7	6085.7	120.78	0.000
2-Way interaction	3	803.1	267.7	5.31	0.040
Soaking time (h)*Sucrose concentration (% w/v)	1	0.2	0.2	0.00	0.949
Soaking time (h)*Sprouting time (days)	1	226.8	226.8	4.50	0.078
Sucrose concentration (% w/v)*Sprouting time (days)	1	576.1	576.1	11.43	0.015
Error	6	302.3	50.4		
Lack-of-fit	3	301.9	100.6	682.02	0.000
Pure error	3	0.4	0.1		
Total	15	33036.1			

Table S8. Analysis of Variance of Antioxidant activity for sucrose as elicitor.

Source	DF	Adj. SS	Adj. MS	F-value	P-value
Model	9	1611.01	179.00	46.80	0.000
Linear	3	1238.86	412.95	107.96	0.000
Soaking time (h)	1	46.51	46.51	12.16	0.013
Sucrose concentration (% w/v)	1	1.20	1.20	0.31	0.596
Sprouting time (days)	1	1191.16	1191.16	311.42	0.000
Square	3	347.44	115.81	30.28	0.001
Soaking time (h)*Soaking time (h)	1	45.44	45.44	11.88	0.014
Sucrose concentration (% w/v)*Sucrose concentration (% w/v)	1	8.78	8.78	2.29	0.181
Sprouting time (days)*Sprouting time (days)	1	293.23	293.23	76.66	0.000
2-Way interaction	3	24.70	8.23	2.15	0.195
Soaking time (h)*Sucrose concentration (% w/v)	1	10.44	10.44	2.73	0.150
Soaking time (h)*Sprouting time (days)	1	7.00	7.00	1.83	0.225
Sucrose concentration (% w/v)*Sprouting time (days)	1	7.27	7.27	1.90	0.217
Error	6	22.95	3.82		
Lack-of-fit	3	22.83	7.61	185.87	0.001
Pure error	3	0.12	0.04		
Total	15	1633.96			

Table S9. Statistical parameters used for the analysis of means by the Tukey test of treatments that used chitosan as an elicitor

	Protein	IVPD	CFT	AOX
Degrees of freedom	15	15	15	15
t student	2.131	2.131	2.131	2.131
MSE	0.52	4.13	329.60	28.53
n	16	16	16	16
HSD	0.39	1.08	9.67	2.84

Table S10. Statistical parameters used for the analysis of means by the Tukey test of treatments that used sucrose as an elicitor

	Protein	IVPD	CFT	AOX
Degrees of freedom	15	15	15	15
t student	2.131	2.131	2.131	2.131
MSE	1.23	10.65	50.40	3.82
N	16	16	16	16
HSD	0.59	1.74	3.78	1.04

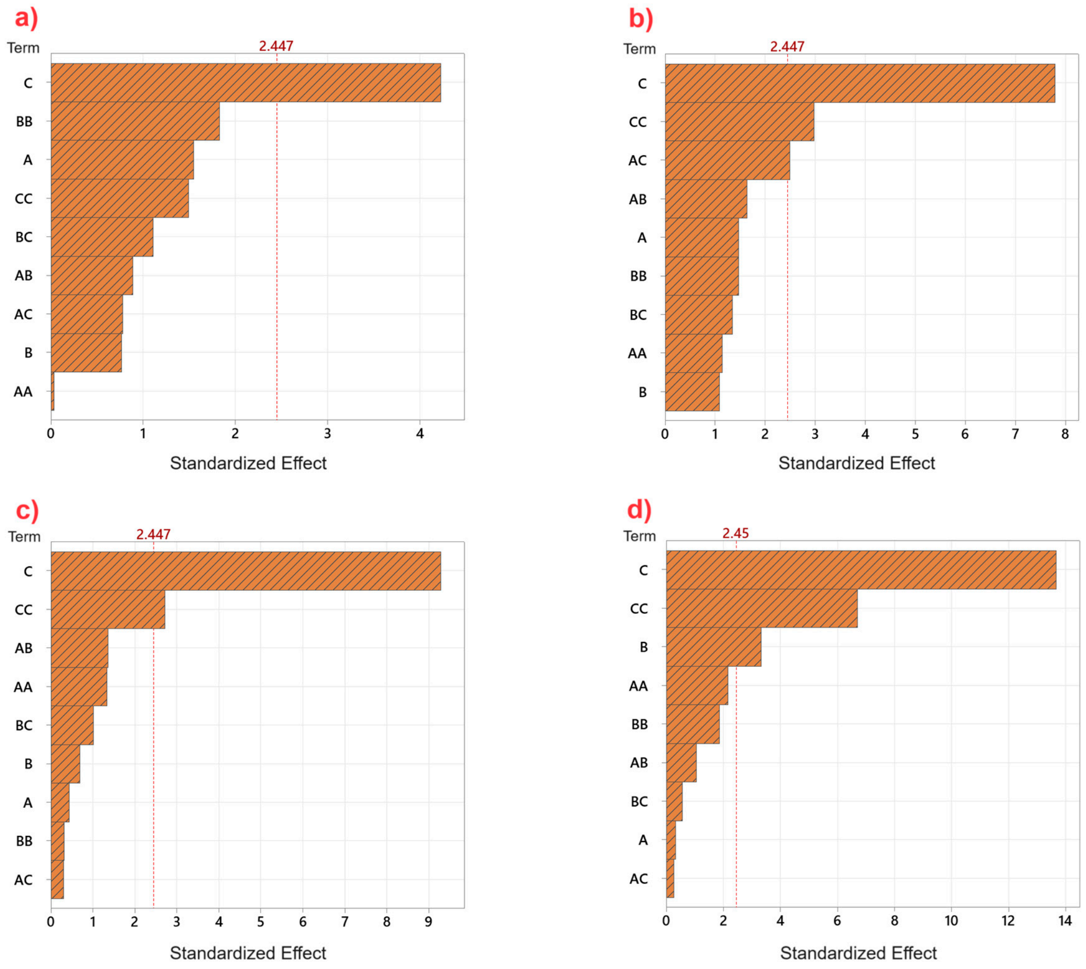

Figure S1. Pareto charts for chitosan as elicitor during chickpea sprouting. a): Protein content, b): *In vitro* protein digestibility (IVPD), c): Total phenolic content (TPC), and d): Antioxidant activity (AOX). A: Soaking time, B: Chitosan concentration, and C: Sprouting time.

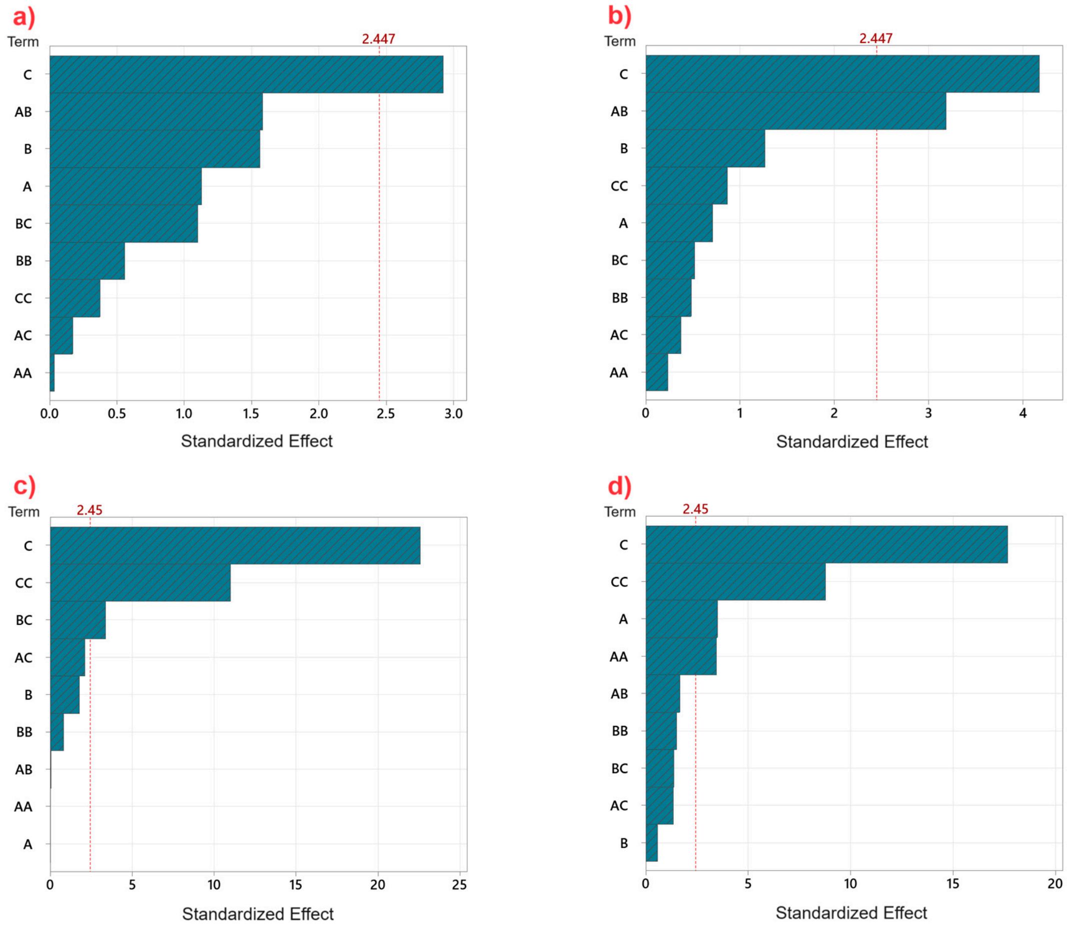

Figure S2. Pareto charts for sucrose as elicitor during chickpea sprouting. a): Protein content, b): *In vitro* protein digestibility (IVPD), c): Total phenolic content (TPC), and d): Antioxidant activity (AOX). A: Soaking time, B: Sucrose concentration, and C: Sprouting time.

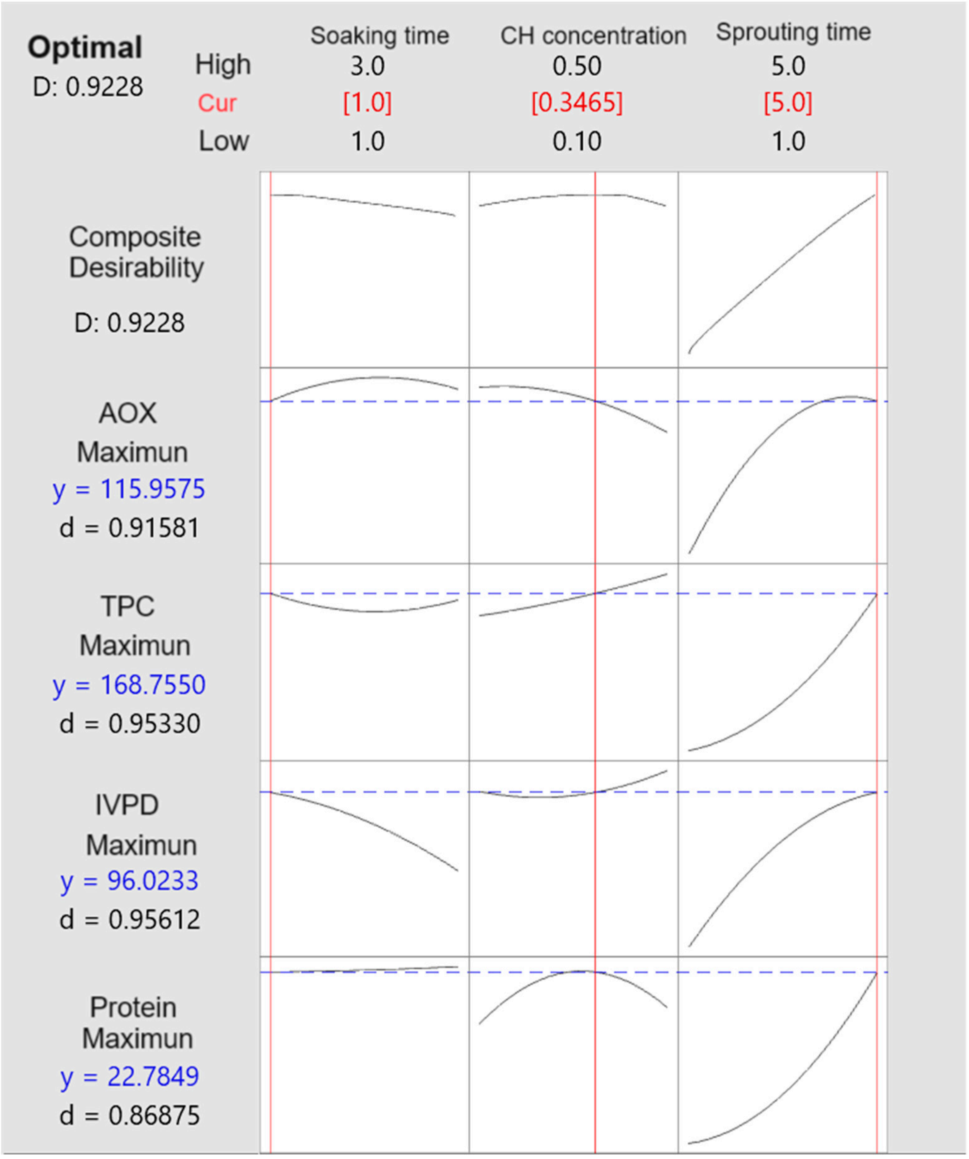

Figure S3. Optimal conditions for chitosan (CH) as elicitor in chickpea germination.

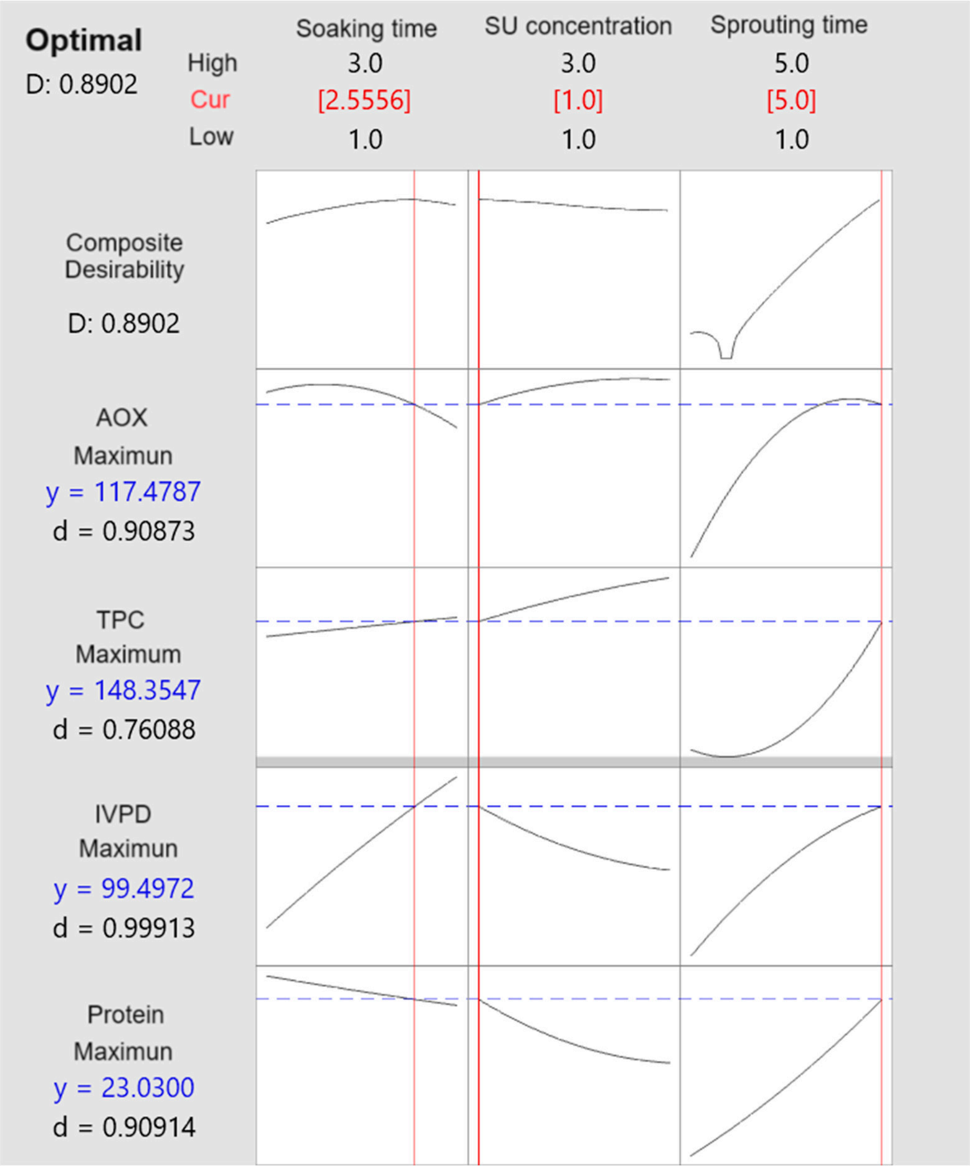

Figure S4. Optimal conditions for sucrose (SU) as an elicitor in chickpea germination.
